# Molecular Epidemiology, Phylogeography, and Recombination Dynamics of PRRSV-2 Sublineage L1C in Mainland China

**DOI:** 10.3390/v18090952

**Published:** 2026-08-31

**Authors:** Jiyu Zhang, Weisheng Wu, Zixuan Wang, Lijun Wang, Mengjuan Yang, Guihong Zhang, Shaoqin Wu

**Affiliations:** 1 Shenzhen Kingkey Smart Agriculture Times Co., Ltd., Shenzhen 518001, China; wuweisheng8910@126.com (W.W.); kakawzx@163.com (Z.W.); lijuning0927@163.com (L.W.); yangmjmj@163.com (M.Y.); 2 Guangdong Provincial Key Laboratory of Zoonosis Prevention and Control, College of Veterinary Medicine, South China Agricultural University, Guangzhou 510642, China; zhangjiyu@stu.scau.edu.cn

**Keywords:** porcine reproductive and respiratory syndrome virus, sublineage L1C, molecular epidemiology, phylodynamics, recombination detection

## Abstract

Since NADC30-like PRRSV-2 was first detected in Henan Province in 2012, it has continued to spread and has become one of the predominant PRRSV-2 groups in mainland China. However, the long-term spatiotemporal dynamics, interprovincial transmission patterns, transmission drivers and recombination associated evolutionary features of sublineage L1C (L1C; NADC30-like) remain incompletely resolved. Here, we analysed 9541 quality-controlled lineage 1 ORF5 sequences from 12 countries, including ORF5 sequences from 62 laboratory-derived complete genomes assigned to L1C. Globally, 3787 sequences were classified as L1C. Among the 1648 lineage 1 sequences from China, 1362 were assigned to L1C, accounting for 82.65% of Chinese lineage 1 sequences. Phylodynamic analysis dated the global common ancestor of L1C to around 2002 and suggested that strains circulating in mainland China were likely introduced from US-related strains around 2008. Before the African swine fever (ASF) outbreak, inferred interprovincial transmission links were concentrated in a limited number of key provinces. During the early ASF period, observable links decreased, but they subsequently recovered and expanded across more provinces. Transmission-driver analysis suggested that pig inventory and pig output were associated with stronger inferred interprovincial L1C transmission links, whereas geographic distance was associated with a spatial-decay effect. Whole-genome recombination analysis revealed extensive recombination signals in L1C genomes involving other PRRSV-2 lineages. Among inter-lineage associations, L8E (HP-PRRSV/JXA1-like) was the most frequently implicated background, followed by L5 and L3. These findings provide systematic evidence for the persistent prevalence, regional transmission and recombination-driven evolution of L1C in mainland China, and support molecular surveillance, regional risk warning and optimization of PRRSV-2 control strategies.

## 1. Introduction

Porcine reproductive and respiratory syndrome virus (PRRSV) is an enveloped, positive-sense single-stranded RNA virus in the order Nidovirales, family Arteriviridae and genus Betaarterivirus [1]. PRRSV infection mainly causes reproductive failure in sows and respiratory disease in piglets and growing pigs, making it one of the most economically important viral pathogens of the global swine industry [2]. PRRSV has traditionally been divided into two genotypes, PRRSV-1 (European type) and PRRSV-2 (North American type), which correspond to Betaarterivirus suid 1 and Betaarterivirus suid 2 under the current ICTV taxonomy. PRRSV-1 is mainly distributed in Europe and is represented by the Lelystad strain, whereas PRRSV-2 predominates in North America and Asia and is represented by VR-2332. The two genotypes share approximately 60% nucleotide identity across the whole genome [3,4]. The PRRSV genome is approximately 15 kb in length and comprises 11 open reading frames (ORF), including ORF1a, ORF1b, ORF2a, ORF2b, ORF3, ORF4, ORF5a, ORF5, ORF6, ORF7, and a short transframe (TF) ORF in the nsp2 region [5]. Because ORF5 is highly variable, it has been widely used for studies of PRRSV genetic diversity, molecular epidemiology and lineage classification [6]. Early ORF5-based studies classified PRRSV-2 into nine major lineages [7], whereas a recent global analysis refined this framework into 11 lineages and 21 sublineages [8]. In China, PRRSV-2 is represented mainly by lineages L1, L3, L5 and L8. NADC30-like PRRSV has continued to circulate since it was reported in China in 2012 and has become one of the predominant PRRSV-2 groups detected in the country [9,10,11,12]. Nevertheless, the long-term epidemic history of L1C in China remains incompletely resolved.

Previous studies have also demonstrated that the spread and regional persistence of HP-PRRSV sublineage 8.7 were shaped by multiple epidemiological and spatial factors, including lineage introduction history, animal movement patterns, and regional transmission connectivity [13]. Similarly, phylogeographic analyses of PRRSV-2 in the United States have shown that incorporating animal movement networks can improve the understanding of viral dissemination pathways, highlighting the importance of host population structure and spatial contact networks in shaping PRRSV transmission dynamics [14]. Furthermore, the African swine fever (ASF) outbreak in China caused substantial changes in swine production systems, animal movement patterns, and biosecurity practices, potentially affecting the epidemiological context of PRRSV transmission [15]. Therefore, these epidemiological and spatial factors should be considered when investigating the transmission dynamics and regional expansion of PRRSV-2 L1C in mainland China, where large-scale pig production, extensive animal transportation networks, and heterogeneous regional production structures may similarly influence viral spread and evolutionary trajectories.

Recombination is another major contributor to PRRSV genetic diversity. Similarly to other RNA viruses, error-prone replication contributes to mutation accumulation and PRRSV evolutionary diversification [16]. Recombination involving wild-type viruses and vaccine-related strains, including JXA1- and CH-1R-related viruses, has been reported and may influence viral evolutionary trajectories, vaccine effectiveness, or generate viruses with altered biological characteristics [17,18,19]. However, despite the widespread circulation and continuous diversification of NADC30-like PRRSV-2, the genomic characteristics and recombination patterns of L1C viruses circulating in mainland China remain insufficiently characterized, particularly during the recent period of 2023–2025.

To address these knowledge gaps, we integrated global and mainland Chinese lineage 1 ORF5 sequences to characterize the temporal dynamics, geographic distribution, and inferred transmission patterns of PRRSV-2 L1C. In parallel, whole-genome analyses were performed on laboratory-derived L1C viruses collected during 2023–2025 to investigate recombination patterns and the genomic regions potentially contributing to L1C diversification. This study aimed to clarify the evolutionary status of L1C in mainland China, reconstruct changes in inferred interprovincial dissemination patterns before and after the ASF outbreak, and characterize the genomic distribution of recombination signals involving major PRRSV-2 lineage backgrounds.

## 2. Materials and Methods

### 2.1. Sequence Dataset Collection and Preparation

The sequence dataset analyzed in this study consisted of two components: publicly available PRRSV-2 ORF5 sequences retrieved from GenBank (National Center for Biotechnology Information [NCBI], Bethesda, MD, USA; accessed in July 2025) using NCBI Entrez Direct (EDirect; NCBI, Bethesda, MD, USA), and an in-house dataset of PRRSV-2 whole-genome sequences previously obtained by our laboratory. Public PRRSV-2 ORF5 sequences were retrieved from GenBank. Sequence retrieval was performed using NCBI Entrez Direct with the following query: (“Porcine reproductive and respiratory syndrome virus 2” [Organism] OR “Betaarterivirus suid 2” [Organism] OR PRRSV-2 [All Fields]) AND (ORF5 [Gene] OR ORF5 [All Fields] OR “glycoprotein 5” [All Fields]) AND (“1998” [PDAT]: “2025” [PDAT]). The query retrieved 38,937 candidate records. Subsequently, the data were further filtered. Sequences were retained if they contained the ORF5 coding region, included available sampling year and geographic information, ranged from 540 to 720 nt in length, and contained no more than 1% ambiguous nucleotides (N). Sequences were excluded if they lacked reliable temporal or geographic information, had unclear origins, were annotated as laboratory-adapted or vaccine strains, showed incomplete ORF5 coverage, or contained excessive ambiguous bases. Ultimately, approximately 20,000 PRRSV-2 ORF5 sequence records were obtained. The in-house dataset consisted of PRRSV-2 whole-genome sequences previously obtained by our laboratory. These sequences were derived from PRRSV-2-positive clinical samples collected through routine veterinary diagnostic activities and active surveillance in 14 provinces of mainland China between 2023 and June 2025. The specimens included serum, lung tissue, tonsil tissue, and lymph node samples collected from infected pigs.

### 2.2. ORF5-Based Lineage Classification and Selection of L1C Genomes

Lineage and sublineage assignment was performed according to the global ORF5-based PRRSV-2 classification framework proposed by Yim-Im et al. Representative reference sequences corresponding to the established PRRSV-2 lineages and sublineages were included in the ORF5 phylogenetic analysis, and study sequences were assigned according to their clustering within the corresponding reference-supported clades. Detailed information on the complete Yim-Im reference set, comprising 1100 representative ORF5 sequences spanning PRRSV-2 lineages L1–L11, including their Yim-Im labels, strain names, and GenBank accession numbers, is provided in Supplementary Table S1. For downstream analyses, L1C-Others/L1C-unclade and L1C.1–L1C.5 were collectively analysed as L1C. Based on the ORF5 phylogenetic classification results, genomes assigned to L1C were selected for subsequent analyses. A total of 62 PRRSV-2 whole-genome sequences belonging to L1C were included in this study. The whole-genome sequences of the selected L1C genomes were used for genome-wide recombination analysis. In parallel, the corresponding ORF5 sequences were integrated with publicly available ORF5 sequences retrieved from GenBank to construct the PRRSV-2 lineage 1 ORF5 dataset for subsequent molecular epidemiological, phylogenetic, phylodynamic, and spatial-transmission analyses.

### 2.3. Dataset Construction

For construction of the main lineage 1 ORF5 dataset, temporal and geographic metadata were standardized at the sampling-year and country levels, respectively. Sequences were retained when both a reliable sampling year and country of origin were available. After sequence-quality filtering, dereplication, and integration of the 62 in-house sequences, the final lineage 1 ORF5 dataset contained 9541 analytical sequences from 12 countries. Supplementary Table S2 lists the GenBank accession numbers and sampling years of all publicly available L1C ORF5 sequences from mainland China included in this study.

Regarding the construction of datasets for subsequent temporal-scale phylogenetic analysis, sequences lacking specific month or day information were handled differently according to the analysis scale. For the global-scale analysis, sequences with reliable country and sampling-year information were retained. For the mainland China-focused analysis, sequences were required to have provincial geographic information, sampling year, and sampling month; the sampling day was then standardized to the first day of the corresponding month. The resulting datasets with complete spatiotemporal metadata were used for subsequent time-scaled phylogenetic analyses, during which sequences showing substantial deviation from the molecular-clock signal were further excluded (see Section 2.5).

### 2.4. Phylogenetic Analysis

Lineage and sublineage classification of PRRSV-2 ORF5 sequences was performed according to the Yim-Im global ORF5-based PRRSV-2 classification framework. The original GenBank Entrez query retrieved 38,937 candidate PRRSV-2 ORF5 records, of which 34,251 were 540–720 nt in length. After sequence-quality and metadata screening (Section 2.1), the retained sequences (already containing the complete set of 1100 Yim-Im reference sequences spanning lineages L1–L11; Supplementary Table S1) were aligned using MAFFT v7.520 [20] and trimmed with trimAl v1.4.rev22 [21]. An initial maximum-likelihood (ML) tree was constructed using IQ-TREE v2.4.0 [22] to identify lineage 1 sequences based on their phylogenetic clustering with the corresponding Yim-Im lineage 1 reference sequences.

Public lineage 1 sequences were then subjected to further quality filtering, temporal and geographic standardization (Section 2.1 and Section 2.3), and dereplication with CD-HIT-EST v4.8.1 at a 99.9% nucleotide identity threshold [23]. Yim-Im reference sequences were retained as classification anchors. The non-redundant public lineage 1 sequences were combined with the ORF5 sequences extracted from the 62 in-house whole-genome sequences, resulting in a final phylogenetic dataset of 9541 sequences (including the 1100 Yim-Im references) from 12 countries. DQ478308.1 was excluded from this dataset.

The final dataset was realigned with MAFFT and trimmed with trimAl. A second ML tree was inferred using IQ-TREE v2.4.0 to assign sequences to established sublineages and to identify L1C sequences, again using the Yim-Im references as anchors. Sublineages L1C.1–L1C.5 together with sequences designated L1C-Others/L1C-unclade were analysed collectively as L1C. Based on this classification, the 62 in-house genomes were assigned to L1C; their complete genomes were used for recombination analysis, while the corresponding ORF5 sequences were included in subsequent molecular epidemiological, phylogenetic, phylodynamic and spatial-transmission analyses.

For the final tree, ModelFinder selected GTR+F+I+G4 as the best-fitting model according to the Bayesian information criterion [24]. Branch support was assessed with 1000 ultrafast bootstrap replicates and 1000 SH-aLRT replicates. DQ478308.1 was added as an external rooting reference, yielding a tree with 9542 tips. No topological constraints were applied.

### 2.5. Phylodynamics and Spatial-Transmission Reconstruction

The spatiotemporal evolution of PRRSV-2 L1C was analysed using the Nextstrain toolkit (Augur v30.0.0) [25]. Global and mainland China ORF5 datasets were constructed according to the study scale and analysed within this Nextstrain framework. TreeTime v0.11.4 was used for time calibration and estimation of the time to the most recent common ancestor (TMRCA) of internal nodes [26], whereas potential spatial transmission history was reconstructed using augur traits and geographic metadata. Briefly, sequences were quality controlled and filtered with augur filter, and multiple-sequence alignment was performed with augur align calling MAFFT [20]. Maximum-likelihood trees were inferred with augur tree calling IQ-TREE, and time calibration under a molecular-clock model was performed with augur refine calling TreeTime. To reduce the influence of sequences that deviated from the molecular-clock signal, temporal outliers were identified and removed using the clock-filtering option in augur refine. Internal-node dates were inferred from sampling times and the molecular-clock model. For each TMRCA estimate, temporal uncertainty was derived from the uncertainty estimates of ancestral node dates generated by TreeTime within the Nextstrain framework. The 95% uncertainty interval was defined by the lower and upper bounds of the corresponding TreeTime-estimated node-date confidence interval. After a time-resolved phylogenetic tree was obtained, augur traits and geographic metadata were used to reconstruct discrete geographic states of internal nodes. Country was used as the discrete geographic state in the global analysis, and province was used in the mainland China analysis.

To examine changes in the interprovincial transmission network of PRRSV-2 sublineage L1C in mainland China before and after the 2018 ASF outbreak, inferred interprovincial geographic-state transitions were summarized from the time-resolved phylogeny and ancestral-state reconstruction. An interprovincial transition was defined as a branch event in which the province state differed between a parent and child node. The direction of transition was assigned from the province represented by the parent node to that represented by the child node, and the child-node num_date was used as the transition time. Based on transition times, interprovincial geographic-state transitions were divided into four periods: before 2018, 2018–2019, 2020–2021, and 2022–2025.

### 2.6. Analysis of Transmission Drivers

To evaluate potential ecological, geographic, and socioeconomic drivers of L1C spatial spread in mainland China, we used a Bayesian phylogeographic generalized linear model (GLM) framework with Bayesian stochastic search variable selection (BSSVS) for covariate selection and effect estimation [27]. Candidate predictors included pig inventory, marketed pig output, distance from Henan Province, gross domestic product (GDP), annual mean temperature, annual precipitation, and mean altitude. All continuous variables were standardized before modelling. Posterior model outputs were extracted, organized, and visualized using scripts written in Python v3.12.2 and are reported as inclusion probability, conditional effect size, and 95% highest posterior density interval (95% HPD).

### 2.7. Recombination Analysis

Recombination analysis was performed on the 62 laboratory-derived L1C complete genomes (Supplementary Table S3). Three publicly available, lineage-representative complete genomes were included as comparators: QYYZ (L3; JQ308798.1), VR-2332 (L5; AY150564.1) and JXA1 (L8E; EF112445.1). These selected backgrounds are epidemiologically relevant to PRRSV-2 circulation in China and to recombination reported for NADC30-like viruses. The analysis was restricted to these backgrounds and was not an exhaustive screen against every recognized PRRSV-2 lineage. The comparator sequences were not assumed to be the direct biological parents of the analysed viruses.

Recombination screening used seven methods implemented in RDP4 v4.101: RDP, GENECONV, BootScan, MaxChi, Chimaera, SiScan and 3Seq [28]. A candidate event was retained when a signal in a similar genomic interval was supported by at least four methods with broadly consistent breakpoint estimates. Candidate events were further examined for reticulate phylogenetic signals using SplitsTree v4.18.3 and the Neighbor-Net algorithm [29]. Breakpoint coordinates were mapped to the CHsx1401 reference genome (GenBank accession KP861625.1). Putative major- and minor-parent assignments were interpreted as regional sequence relationships relative to the included comparators rather than as identification of direct ancestral viruses.

## 3. Results

### 3.1. Dataset Characteristics of PRRSV-2 Lineage 1 Viruses

**Figure 1:**
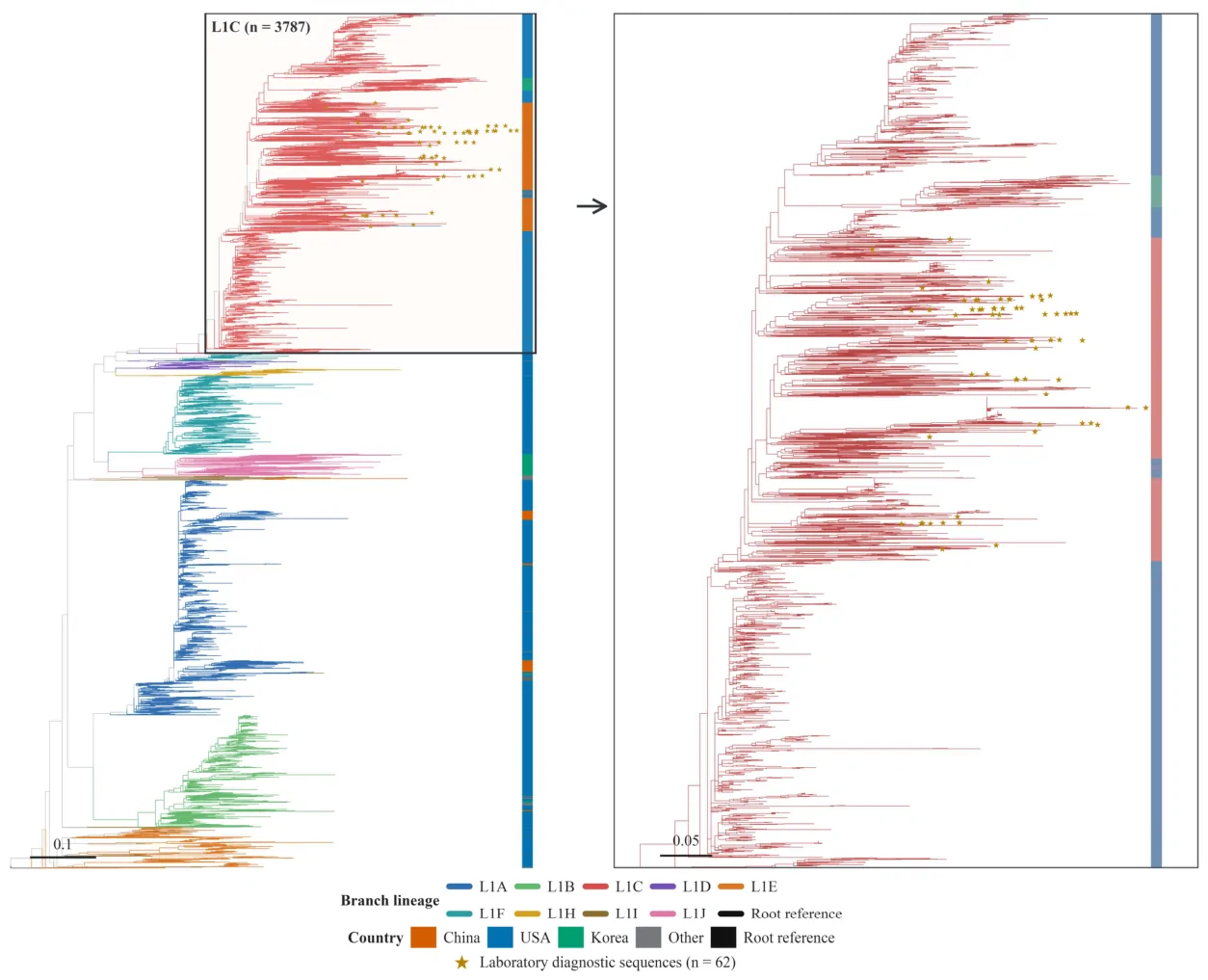
Maximum-likelihood phylogeny of the global PRRSV-2 lineage 1 ORF5 dataset. The tree includes 9541 analytical sequences and the external rooting reference DQ478308.1.

**Figure 2:**
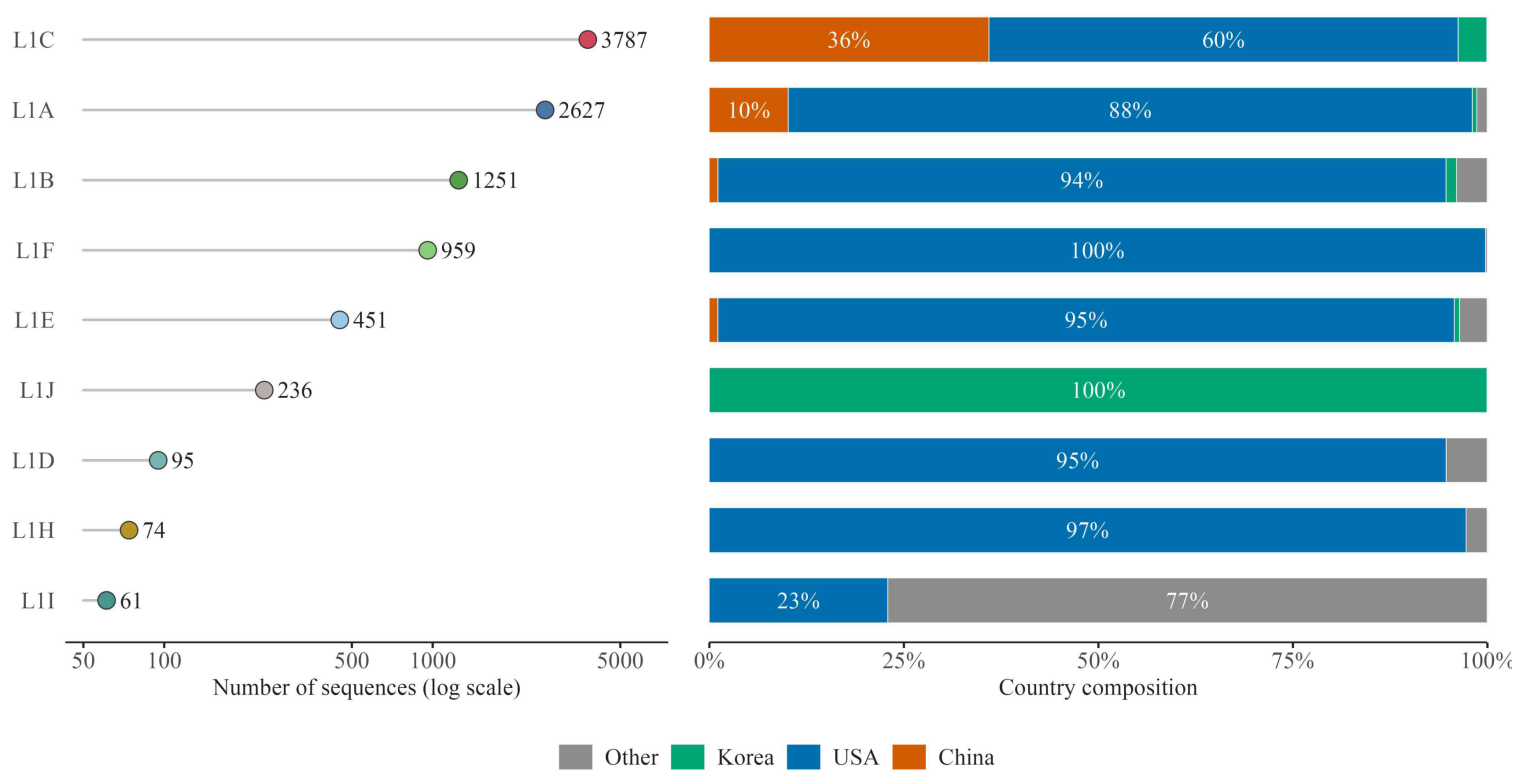
Sampling depth and country composition of the global PRRSV-2 lineage 1 ORF5 dataset. The left panel shows the number of sequences in each lineage 1 sublineage on a logarithmic scale. The right panel shows the country composition within each sublineage. Percentages are displayed for country components accounting for at least 8% of sequences within the respective sublineage.

The final analytical dataset comprised 9541 lineage 1 ORF5 sequences from 12 countries: 9479 public records and 62 laboratory-derived L1C sequences. The maximum-likelihood tree contained 9542 tips after addition of the external rooting reference DQ478308.1 (Figure 1). L1C was the most abundant parent group, comprising 3787 sequences (39.69%), followed by L1A (2627; 27.53%), L1B (1251; 13.11%), L1F (959; 10.05%), L1E (451; 4.73%), L1J (236; 2.47%), L1D (95; 1.00%), L1H (74; 0.78%) and L1I (61; 0.64%). The United States contributed 7325 sequences (76.77%) and China contributed 1648 (17.27%); Korea contributed 411 (4.31%), and the remaining nine countries each contributed fewer than 50 sequences. Among L1C sequences, 2284 originated from the United States (60.31% of L1C), 1362 from China (35.97%), 140 from Korea (3.70%) and one from Canada (0.03%). L1C represented 31.18% of US lineage 1 sequences and 82.65% of Chinese lineage 1 sequences (Figure 2).

### 3.2. Spatiotemporal Distribution of L1C

To characterize the temporal distribution of lineage 1, we grouped sequences into four sampling periods. The 1998–2008, 2009–2013, 2014–2018 and 2019–2025 periods contained 1991 (20.87%), 2545 (26.67%), 3571 (37.43%) and 1434 (15.03%) sequences, respectively. L1C contributed 12, 1644, 1131 and 1000 sequences in these periods, corresponding to 0.60%, 64.60%, 31.67% and 69.74% of all lineage 1 sequences within each period (Table 1).

The final Chinese lineage 1 dataset comprised 1648 sequences, including one L1C sequence collected in 2011 (KU523367). Because temporal composition analysis was restricted to 2012–2025, Table 2 is based on the remaining 1647 sequences, of which 1361 (82.64%) were L1C, followed by L1A (267; 16.21%), L1B (14; 0.85%), and L1E (5; 0.30%). L1C remained dominant across the four periods, accounting for 96.50%, 95.32%, 84.32%, and 63.95% of sequences during 2012–2015, 2016–2018, 2019–2021, and 2022–2025, respectively, whereas L1A increased from 0% to 35.37% over the same intervals (Table 2).

### 3.3. Phylodynamic Analysis of PRRSV-2 L1C

**Figure 3:**
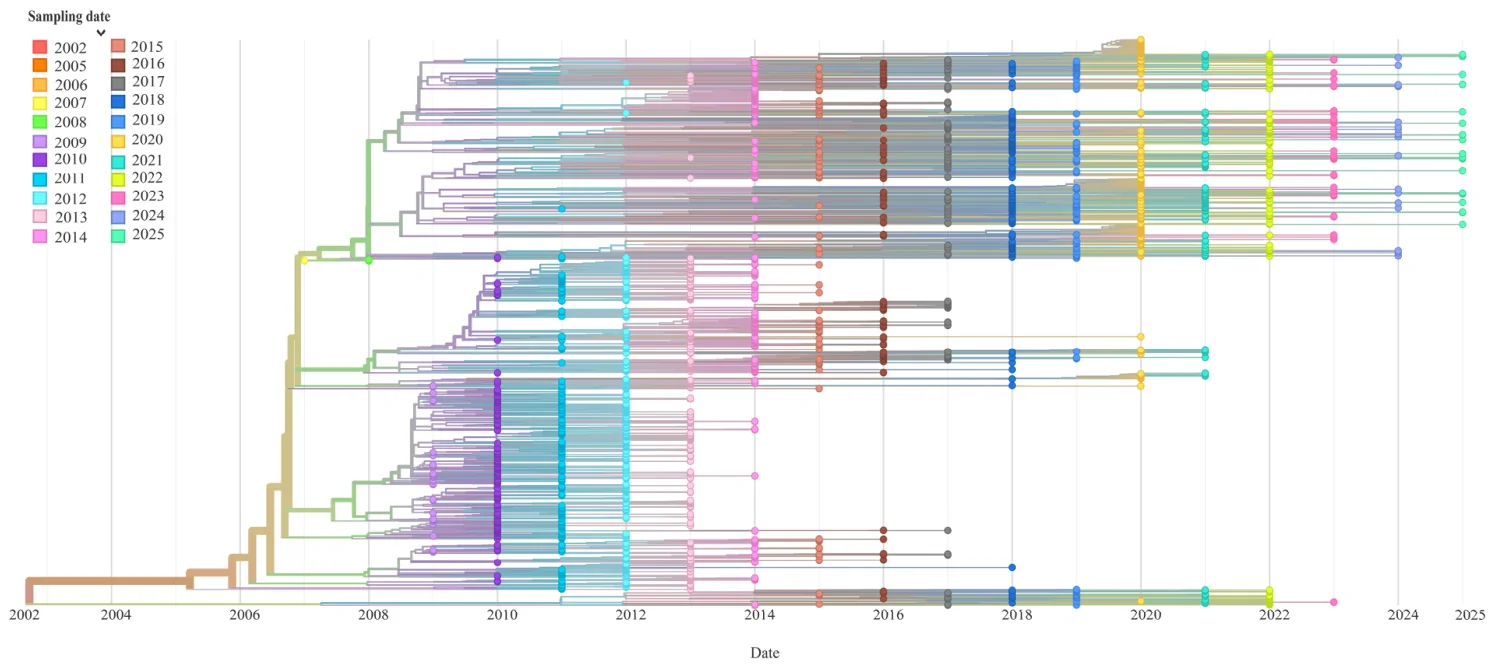
Global time-scaled phylogeny of PRRSV-2 sublineage L1C. Colours of tip nodes indicate the year of sample collection.

**Figure 4:**
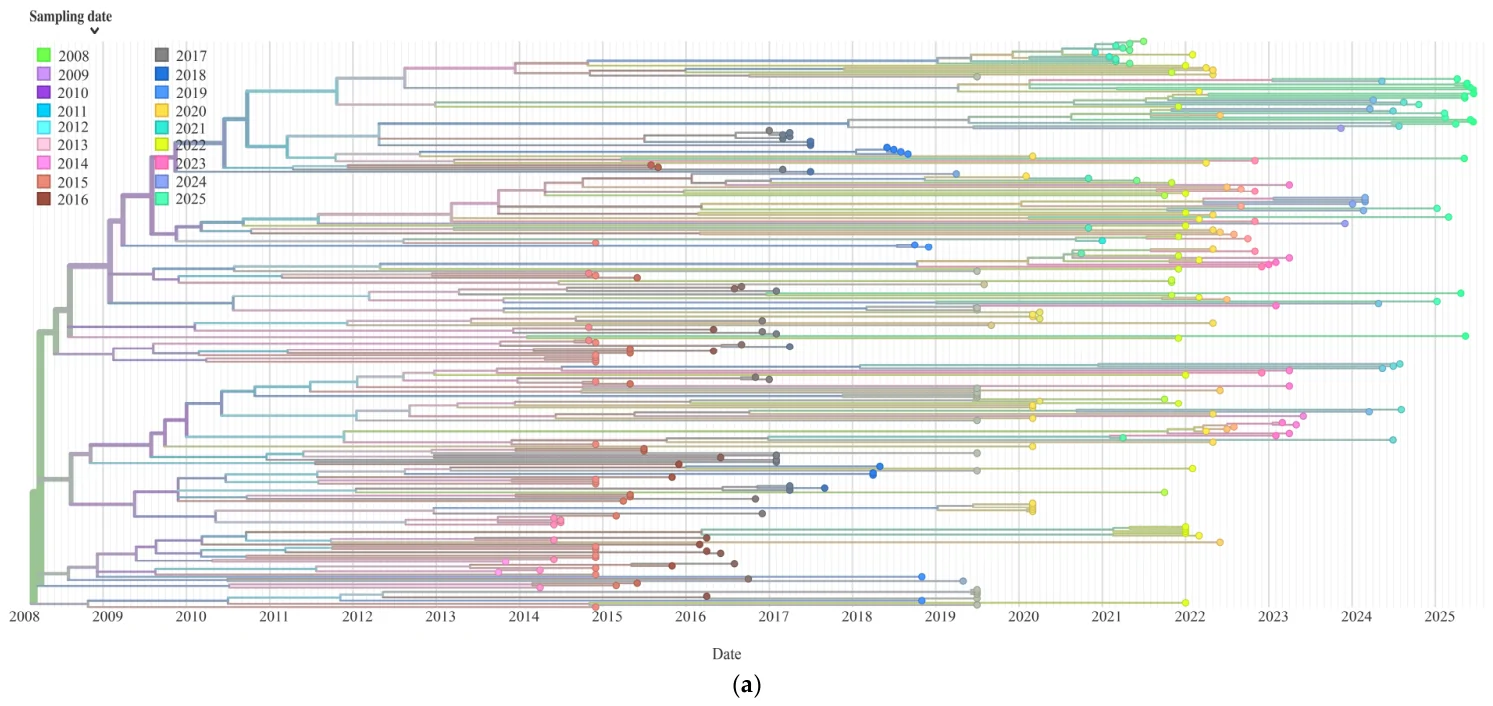

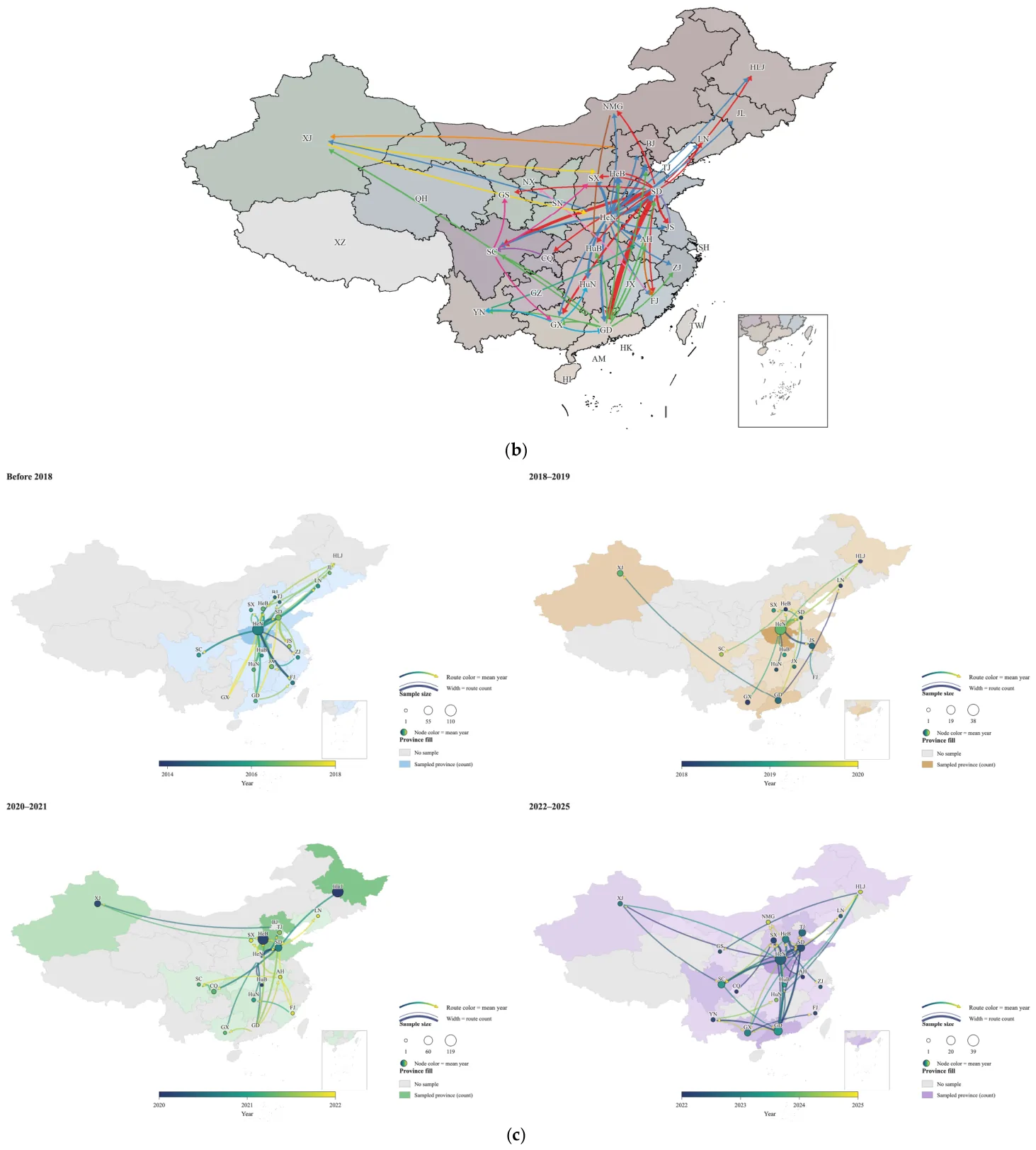
Time-scaled phylogeny and interprovincial transmission reconstruction of L1C strains circulating in mainland China. (**a**) Time-scaled phylogeny of mainland Chinese L1C strains. Colours indicate sampling year and show continuous diversification and detection of multiple branches after 2010. (**b**) Interprovincial transmission routes inferred from geographic-state reconstruction using all mainland Chinese data in this study. Arrows indicate inferred transmission direction, and colours represent source province or transmission route. (**c**) Upper left, pre-2018 baseline period before the ASF outbreak; upper right, 2018–2019 ASF shock and production-decline period; lower left, 2020–2021 restocking and production-recovery period; lower right, 2022–2025 post-recovery normalization period. Arrows indicate province-state transitions inferred from the time-scaled phylogeny and discrete geographic-state reconstruction. Line colour indicates transition time, node size indicates provincial sample count, and provincial fill colour indicates sample abundance in each period, with darker colours representing higher sample numbers.

To characterize the temporal and spatial dynamics of PRRSV-2 L1C evolution, a time-scaled phylogenetic analysis was performed using sequences with complete sampling date and geographic information. The estimated global TMRCA of L1C was approximately 2002 (95% uncertainty interval: 2000–2003). The ancestral geographic state reconstruction suggested a USA-associated origin of the early L1C population, followed by lineage diversification around 2008, with increased branching activity observed during 2008–2011 (Figure 3). These inferred geographic patterns represent model-based estimates rather than direct evidence of specific introduction events. In mainland China, the TMRCA of circulating L1C strains was estimated at approximately 2008, with a 95% uncertainty interval of 2007.8–2010 (Figure 4a). Across its subsequent evolutionary history, the lineage showed sustained branching and substantial genetic diversification. The discrete-trait reconstruction supported a highly interwoven, multicentre geographic pattern (Figure 4b). Inferred links were relatively frequent around Henan, Shandong and Guangdong, and some spanned long geographic distances.

To assess stage-specific changes in the L1C interprovincial transmission network before and after the 2018 ASF outbreak, inferred transmission events were compared across four periods. Before 2018, 43 interprovincial transitions and 27 directed phylogeographic links were detected, involving 166 tip samples and 16 sampled provinces. The network during this baseline period was centred mainly on Henan, with inferred links concentrated in parts of central, northern, eastern, and southern China, indicating a relatively focused outward-spread pattern. During 2018–2019, 23 interprovincial transitions and 16 inferred transmission links were detected, involving 71 tip samples and 14 sampled provinces. The reduction in events and inferred links relative to the pre-2018 period suggests a contraction of observable interprovincial L1C transmission during the initial ASF shock. During 2020–2021, interprovincial transitions increased to 39, inferred transmission links increased to 26, and the number of tip samples reached 315 across 14 sampled provinces. Compared with 2018–2019, both events and inferred links rebounded, and the network again showed stronger cross-regional connectivity. During 2022–2025, 53 interprovincial transitions and 37 inferred transmission links were detected, involving 136 tip samples and 20 sampled provinces. This period had the highest number of inferred links and the widest provincial coverage among the four stages. The network expanded further, with Henan remaining an important node and increased participation from Shandong, Guangdong, Hebei, Heilongjiang, Xinjiang, Chongqing, and Sichuan (Figure 4c).

### 3.4. Transmission Drivers of PRRSV-2 Sublineage L1C in China

Using a Bayesian phylogeographic GLM with BSSVS variable selection, we evaluated statistical associations between candidate predictors and inferred interprovincial L1C transmission risk in mainland China. Pig inventory, distance from Henan and marketed pig output received high model support and had relatively large absolute conditional effect sizes. Pig inventory had the highest inclusion probability (0.95), with a conditional effect size of 0.742 (95% HPD, 0.587–0.897). Distance from Henan had an inclusion probability of 0.92 and a conditional effect size of −0.705 (95% HPD, −0.872 to −0.538). Marketed pig output had an inclusion probability of 0.89 and a conditional effect size of 0.688 (95% HPD, 0.527–0.849). These results indicated that pig inventory and marketed pig output were positively associated with inferred interprovincial L1C transmission risk, whereas distance from Henan was negatively associated with inferred transmission risk.

**Figure 5:**
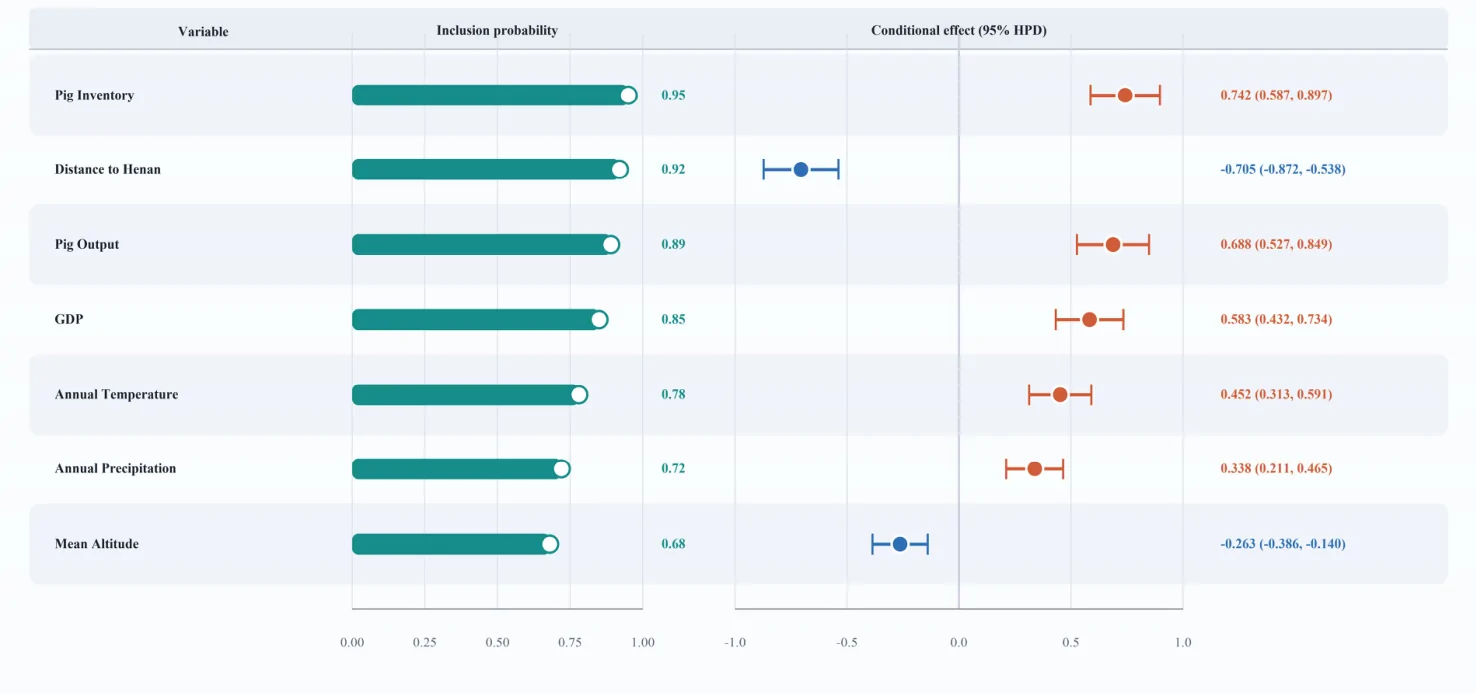
Generalized linear model assessment of candidate covariates associated with inferred L1C transmission intensity in mainland China. The left panel shows inclusion probabilities, and the right panel shows conditional effects with 95% highest posterior density intervals. Positive values indicate positive associations with inferred transmission intensity, whereas negative values indicate negative associations.

GDP also showed high model support, with an inclusion probability of 0.85 and a conditional effect size of 0.583 (95% HPD, 0.432–0.734). By contrast, annual temperature, annual precipitation and mean altitude had smaller absolute conditional effect sizes. Their inclusion probabilities were 0.78, 0.72 and 0.68, respectively, and their conditional effect sizes were 0.452 (95% HPD, 0.313–0.591), 0.338 (95% HPD, 0.211–0.465) and −0.263 (95% HPD, −0.386 to −0.140) (Figure 5). Annual temperature and annual precipitation were positively associated with inferred transmission risk, whereas mean altitude was negatively associated with inferred transmission risk.

### 3.5. Genome-Wide Characterization of Recombination Patterns Among L1C Genomes

**Figure 6:**
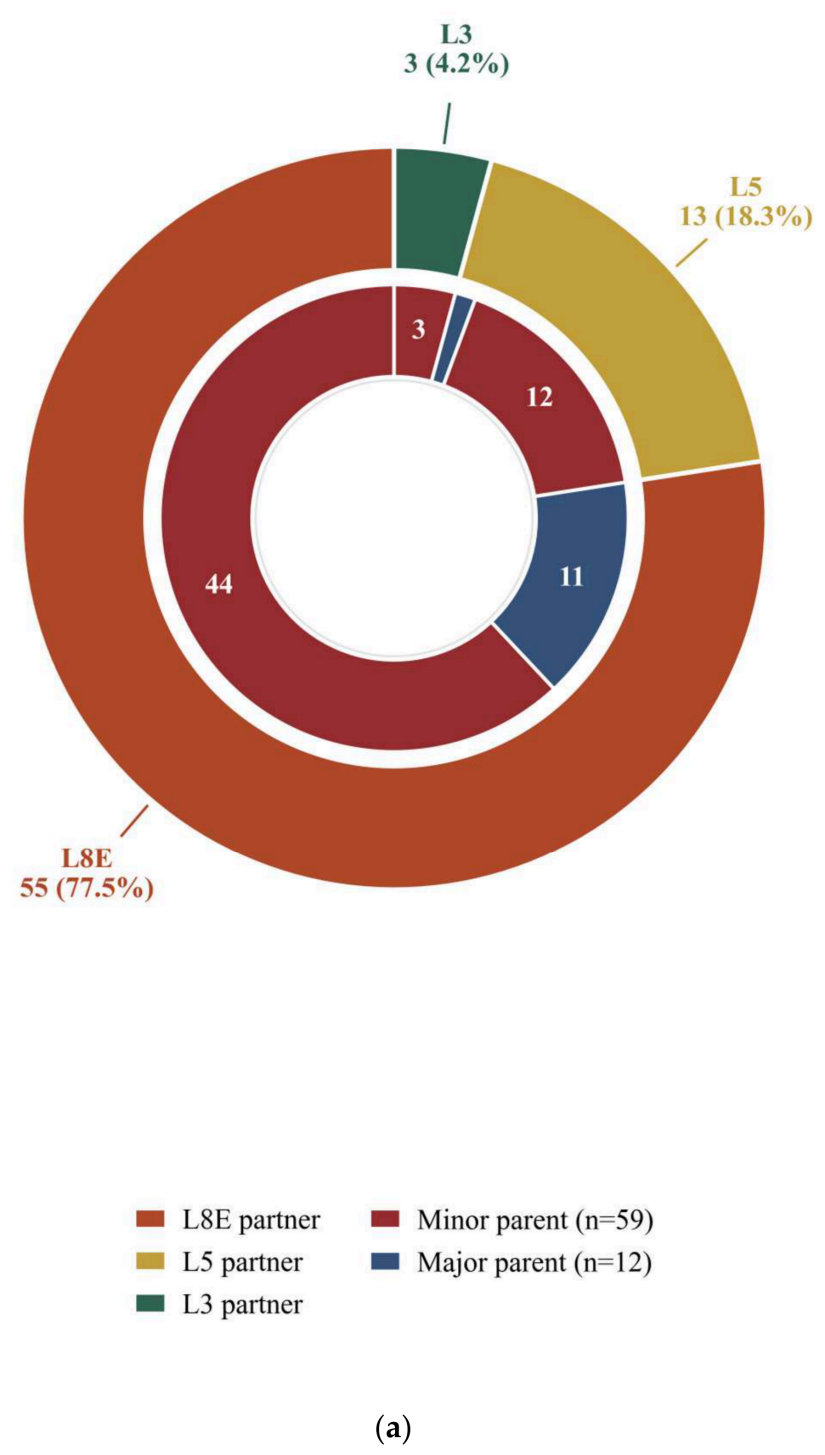

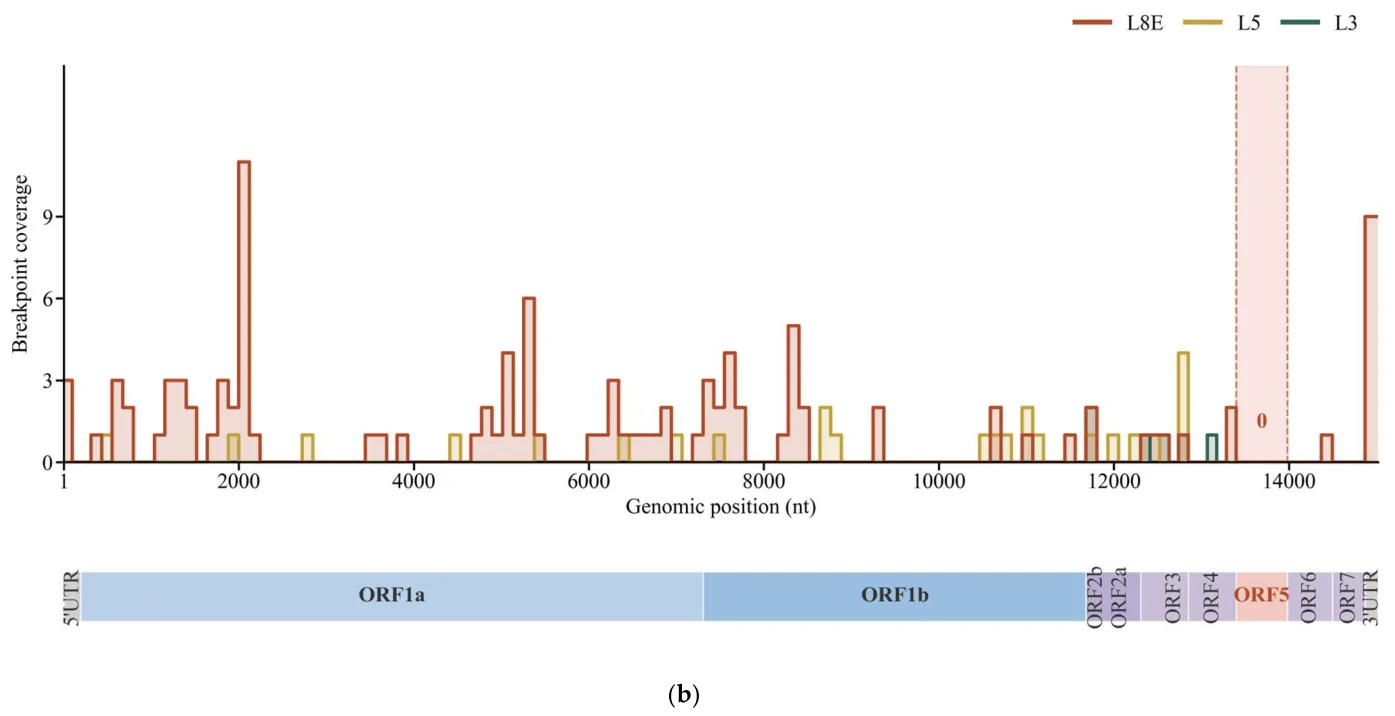
Whole-genome recombination signals detected in 62 laboratory-derived L1C genomes. (**a**) Inter-lineage recombination structure of PRRSV-2 L1C-associated recombination events. (**b**) Genomic coverage distribution of inter-lineage recombination breakpoints in CHsx1401 (KP861625.1).

Among statistically supported RDP events, we quantified heterologous partners and their parental roles for inter-lineage recombination. Only events in which an L8E, L5 or L3 sequence was assigned as the minor or major parent were retained (*n* = 71). L8E was the dominant heterologous partner (55/71, 77.5%), followed by L5 (13/71, 18.3%) and L3 (3/71, 4.2%). Heterologous lineages predominantly acted as minor parents (59/71, 83.1%) rather than major parents (12/71, 16.9%). Specifically, L8E contributed 44 minor-parent and 11 major-parent assignments; L5 contributed 12 and 1; and all three L3 events involved L3 as the minor parent. These findings indicate that inter-lineage recombination in L1C-related genomes is mainly driven by insertion of heterologous donor fragments—particularly of L8E origin—into an L1C genomic backbone (Figure 6a).

To determine the genomic distribution of inter-lineage recombination breakpoints associated with the L1C lineage, we mapped 71 inter-lineage recombination events (totaling 142 breakpoints) to the reference genome CHsx1401 (KP861625.1) and analyzed them based on mutually exclusive genomic intervals. Breakpoints were highly enriched in regions encoding non-structural proteins: ORF1a contained 71 breakpoints (50.0%) and ORF1b contained 35 (24.6%), together accounting for 74.6% (106/142) of all breakpoints. Among regions encoding structural proteins, ORF3 showed a relatively high frequency (13/142, 9.2%), while ORF2 and ORF4 accounted for 7 (4.9%) and 3 (2.1%) breakpoints, respectively, and ORF6 contributed a single breakpoint (1/142, 0.7%). Breakpoints were also detected in UTR regions (3′UTR: 9, 6.3%; 5′UTR: 3, 2.1%). Stratification by heterologous parent revealed that L8E-associated breakpoints were the most frequent (110/142, 77.5%), followed by L5 (26/142, 18.3%) and L3 (6/142, 4.2%) (Figure 6b).

## 4. Discussion

PRRSV-2 lineage 1 continues to circulate in major swine-producing regions worldwide and has diversified into multiple sublineages with distinct geographic distributions. Among these sublineages, L1C (NADC30-like PRRSV) has become an important molecular surveillance target due to its widespread circulation, ongoing diversification, and reported recombination events [30]. Since its emergence in China around 2012, NADC30-like PRRSV has spread across multiple provinces and become one of the predominant PRRSV-2 groups circulating in mainland China [31]. The detection of L1C variants in the United States and Korea further indicates that this sublineage has achieved broader international distribution [32,33]. In the present study, analysis of 9541 global lineage 1 ORF5 sequences from 12 countries further supported the widespread distribution and epidemiological importance of L1C. L1C represented a major component of lineage 1 diversity and showed particularly high predominance among Chinese lineage 1 sequences, highlighting its epidemiological importance and continued diversification in mainland China.

Time-scaled phylogenetic analysis estimated the global L1C TMRCA at approximately 2002 and the TMRCA of the mainland Chinese L1C population at approximately 2008. The estimated ancestral date of Chinese L1C predates the first formal recognition of NADC30-like PRRSV in China and may reflect earlier unsampled circulation or limited surveillance before routine molecular monitoring. Previous studies have suggested that Chinese NADC30-like viruses are genetically related to North American NADC30-like strains, supporting the possibility of introduction followed by subsequent diversification in China [34]. However, node-date and ancestral-state estimates are influenced by sampling density, temporal signal, and model assumptions and should not be interpreted as direct evidence of a specific introduction event. Chinese L1C sequences collected from different years were interspersed throughout the time-scaled phylogeny rather than forming a simple chronological cluster. This pattern is more consistent with sustained circulation and parallel diversification than with a single short-lived outbreak. Long-term circulation of PRRSV, together with its rapid evolutionary dynamics and frequent recombination, may contribute to the accumulation of genetic diversity and increase the complexity of molecular surveillance and control strategies [35].

Spatial-transmission analysis further suggested that L1C dissemination in mainland China did not follow a simple single-centre expansion pattern. Instead, the inferred transmission network showed a multicentre structure, with Henan, Shandong, and Guangdong appearing as relatively important nodes. These provinces represent major regions of swine production and regional circulation in China, and their connectivity within the inferred network may reflect the combined effects of host population size, production intensity, geographic proximity, and regional livestock circulation patterns. Previous molecular epidemiological studies have demonstrated that PRRSV-2 populations in China exhibit complex spatial distributions and regional genetic differentiation, reflecting the combined influence of viral evolution, production systems, and regional epidemiological conditions [36,37]. Although different PRRSV-2 lineages may have distinct evolutionary histories and geographic patterns, these findings highlight the importance of considering spatial structure and host population characteristics when interpreting PRRSV dissemination. Lineage-specific phylogeographic analyses therefore provide an effective approach for understanding how different PRRSV-2 populations spread within complex swine production systems.

Temporal changes in the inferred L1C transmission network coincided with major disruptions and subsequent restructuring of the swine production system during and after the African swine fever (ASF) outbreak. Before 2018, the inferred L1C network showed relatively strong connectivity involving regions such as Henan, which may be related to its intensive swine production capacity and regional circulation characteristics. Following the ASF outbreak, reductions in pig inventory, changes in regional production and movement patterns, and enhanced biosecurity practices may have influenced the observed structure of L1C transmission links. With the recovery of swine production, restocking activities, and regional circulation after 2020, inferred L1C transmission links increased again and showed a more complex multicentre structure involving multiple provinces. These temporal changes are consistent with the possibility that major disruptions to swine production systems may influence PRRSV-2 spatial dynamics [38,39]. However, these temporal patterns should be interpreted cautiously, as the observed association does not establish a direct causal relationship between ASF-related changes and L1C transmission dynamics. The reconstructed interprovincial links represent geographic-state transitions inferred from viral phylogenies rather than direct observations of pig movements or transmission chains. In addition, differences in sampling intensity and sequence availability among provinces and time periods may influence inferred connectivity patterns and the apparent importance of specific regions, including Henan. Therefore, the inferred network should be regarded as a phylogeographic reconstruction of evolutionary relationships among sampled viruses rather than direct evidence of actual transmission routes.

In this study, interprovincial L1C transmission patterns were evaluated by integrating phylogeographic reconstruction with regional demographic, economic, and environmental variables. Pig inventory and marketed pig output showed positive associations with inferred transmission patterns, suggesting that regions with larger swine populations and greater production activity may provide more opportunities for viral persistence and dissemination. Previous studies have also demonstrated heterogeneous geographic distributions of PRRSV-2 lineages in China [40,41]. Geographic distance showed a negative association with L1C diffusion, indicating spatial attenuation of inferred viral exchange, consistent with previous observations that PRRSV transmission and genetic relatedness may decrease with increasing geographic distance [42]. GDP showed a positive association but should be interpreted cautiously because it represents a composite indicator reflecting multiple socioeconomic processes, including industrial development, transportation infrastructure, livestock production, and surveillance capacity. In contrast, climatic and topographic variables showed weaker effects, suggesting that production-related factors may contribute more strongly to contemporary L1C transmission patterns, although environmental conditions may influence PRRSV epidemiology under specific production contexts [43]. Importantly, these associations represent statistical relationships between candidate factors and inferred transmission patterns rather than direct causal effects. In addition, reconstructed transmission links reflect phylogeographic relationships among sampled viruses rather than direct pig movements or transmission chains, and differences in sampling intensity and sequence availability among regions and periods may influence the inferred connectivity patterns.

The parental composition of inter-lineage recombination highlights the genomic plasticity of L1C (NADC30-like) viruses in China. L8E viruses were identified as the predominant heterologous partners in the analyzed dataset, consistent with the frequent co-circulation and reported recombination between NADC30-like and HP-PRRSV-related viruses in China. In comparison, L5 and L3 contributed fewer inferred recombination associations, suggesting that their contribution to L1C evolution may be less frequent within the sampled population. The predominance of heterologous lineages as inferred minor parents indicates a pattern in which L1C viruses maintain their genomic backbone while acquiring genomic fragments from other circulating lineages, contributing to PRRSV-2 genetic diversification [44]. Inferred recombination breakpoints were mainly located in replicase-associated regions, particularly ORF1a and ORF1b, consistent with previous reports that PRRSV recombination frequently involves non-structural genomic regions [45]. However, these inferred parental relationships and breakpoint patterns should be interpreted cautiously, as computational recombination methods reflect sequence-based evidence rather than direct biological donor viruses and may be affected by sampling and reference availability.

## 5. Conclusions

Overall, this study characterized the evolutionary dynamics of PRRSV-2 L1C in mainland China within the global lineage 1 context by integrating temporal phylogenetic analysis, interprovincial phylogeographic reconstruction, transmission-associated factor analysis, and whole-genome recombination assessment. L1C has remained the predominant lineage 1 sublineage in China and has undergone long-term circulation and diversification rather than representing a single recent introduction or short-lived outbreak. Temporal reconstruction revealed changes in the inferred L1C transmission network before and after the African swine fever (ASF) outbreak, suggesting that large-scale disruptions and subsequent restructuring of the swine production system may influence the spatial dynamics of PRRSV-2. The inferred dissemination patterns were associated with host population size, regional circulation intensity, and geographic proximity, highlighting the contribution of production-related and spatial factors to L1C spread. Whole-genome recombination analysis further revealed frequent inter-lineage recombination involving L1C and the selected comparator lineages, including L8E, L5, and L3. Among these backgrounds, L8E represented the predominant recombination-associated lineage, whereas L5 and L3 contributed less frequently. These findings improve understanding of how persistent circulation, spatial transmission processes, and genetic exchange jointly shape the evolution of L1C in mainland China.

These findings deepen understanding of the persistent circulation and genetic evolution of L1C in mainland China. They also indicate that surveillance of this sublineage should not be limited to single-outbreak investigations, ORF5 typing or genome comparisons of individual strains. Instead, continuous sampling, whole-genome sequencing, time-scaled phylogenetic analysis, spatial-transmission reconstruction, pig-movement data and recombination surveillance should be integrated to dynamically assess transmission risk and genetic-variation trends. This study provides systematic evidence for identifying key transmission regions, optimizing regionalized control strategies and early warning for emerging recombinant strains, and offers a new perspective on the long-term maintenance and evolution of PRRSV-2 in China’s complex swine-production system.

## Figures and Tables

**Table 1:** Temporal distribution of global PRRSV-2 lineage 1 and L1C sequences.

Sampling Period	All Lineage 1, *n*	Dataset Share, %	L1C, *n*	L1C Within Period, %
1998–2008	1991	20.87	12	0.60
2009–2013	2545	26.67	1644	64.60
2014–2018	3571	37.43	1131	31.67
2019–2025	1434	15.03	1000	69.74
Total	9541	100.00	3787	39.69

**Table 2:** Temporal composition of mainland Chinese PRRSV-2 lineage 1 sequences sampled during 2012–2025.

Sampling Period	Total, *n*	L1C, *n* (%)	L1A, *n* (%)	L1B, *n* (%)	L1E, *n* (%)
2012–2015	143	138 (96.50)	0 (0.00)	5 (3.50)	0 (0.00)
2016–2018	406	387 (95.32)	14 (3.45)	4 (0.99)	1 (0.25)
2019–2021	657	554 (84.32)	97 (14.76)	4 (0.61)	2 (0.30)
2022–2025	441	282 (63.95)	156 (35.37)	1 (0.23)	2 (0.45)
Total	1647	1361 (82.64)	267 (16.21)	14 (0.85)	5 (0.30)

## Data Availability

The representative reference sequences used for ORF5-based lineage and sublineage assignment are listed in Supplementary Table S1. The publicly available Chinese PRRSV-2 lineage 1C ORF5 sequences analyzed in this study are available from the National Center for Biotechnology Information (NCBI) GenBank database, and their accession numbers are provided in Supplementary Table S2. The laboratory-derived PRRSV-2 lineage 1C complete genome sequences generated in this study have been deposited in GenBank under accession numbers PZ822893–PZ822954; their associated metadata are provided in Supplementary Table S3.

## Supplementary Materials

The following supporting information can be downloaded at: https://www.mdpi.com/article/10.3390/v18090952/s1. Supplementary Table S1 contains the Yim-Im labels, strain names, and GenBank accession numbers of the representative reference sequences used for ORF5-based lineage and sublineage assignment. Supplementary Table S2 contains the GenBank accession numbers of all publicly available Chinese L1C ORF5 sequences included in this study. Supplementary Table S3. GenBank accession numbers and metadata of laboratory-derived PRRSV-2 L1C complete genomes analyzed in this study.
